# The Efficacy of Short-Term Toe Resistance Training in Chronic Hemodialysis Patients with Type 2 Diabetes

**DOI:** 10.3390/healthcare11010137

**Published:** 2023-01-01

**Authors:** Hiroaki Kataoka, Nobuyuki Miyatake, Naoko Matsuda, Yasuaki Hikasa, Naomi Kitayama, Shion Nagai, Satoshi Tanaka

**Affiliations:** 1 Department of Physical Therapy, Faculty of Health Sciences, Okayama Healthcare Professional University, Okayama 700-0913, Japan; 2 Department of Hygiene, Faculty of Medicine, Kagawa University, Takamatsu 761-0793, Japan; 3 Department of Rehabilitation, Osafune Clinic, Okayama 701-4264, Japan; 4 Department of Rehabilitation, Obata Medical Clinic, Okayama 708-0806, Japan; 5 Rehabilitation Center, KKR Takamatsu Hospital, Takamatsu 760-0018, Japan; 6 Department of Physical Therapy, Faculty of Health and Welfare, Prefectural University of Hiroshima, Hiroshima 723-0053, Japan

**Keywords:** chronic hemodialysis, type 2 diabetes, toe pinch force, toe resistance training, randomized controlled trial

## Abstract

Resistance training is effective in chronic hemodialysis patients with type 2 diabetes mellitus, but its effect on toe pinch force (TPF) is unknown. This study was a randomized controlled trial conducted at three hospitals to investigate the effect of short-term toe resistance training on TPF in chronic hemodialysis patients with type 2 diabetes. The patients were randomly allocated to intervention (performed aerobic exercise and four toe resistance training exercises) and control (performed aerobic exercise only) groups. After 2 weeks of exercise intervention program, evaluations of TPF and clinical parameters were performed. In addition, the rate of retention of exercise therapy was assessed 6 months after the exercise intervention program was completed. After the exercise intervention program, TPF was significantly higher in the intervention group than in the control group. The intervention group had a significantly higher rate of continuation of exercise therapy. Two weeks of toe resistance training significantly increased the TPF in chronic hemodialysis patients with type 2 diabetes. Toe resistance training was shown to be an effective training method for continuing exercise therapy. Toe resistance training is recommended in clinical practice for chronic hemodialysis patients with type 2 diabetes.

## 1. Introduction

Exercise therapy is recommended as an important treatment modality in chronic hemodialysis patients with type 2 diabetes. However, hemodialysis requires patients to lie in bed for approximately 4 h per treatment, 3 days per week. A combination of factors, including a high incidence of general malaise, fatigue, and hypotension after hemodialysis, is known to reduce the amount of physical activity [1]. Decreased physical activity has a negative impact on cardiopulmonary function, and chronic hemodialysis patients are reported to have reduced exercise tolerance comparable to patients with cardiac or respiratory disease [2]. Furthermore, a study investigating motor function in chronic hemodialysis patients reported that motor function in older patients was 50% lower than that in healthy older adults [3]. In light of the above, exercise therapy is actively used in chronic hemodialysis patients with type 2 diabetes, and is reported to improve exercise tolerance [4,5,6,7], walking ability [4,5], and physical quality of life [4,5,6]. In contrast, a survey on exercise habits conducted by the Japanese Society for Dialysis Therapy on chronic hemodialysis patients reported that about 60–80% of patients answered that they “have no exercise habit” or “have little exercise habit [8]”, indicating that lack of exercise is a serious issue. Beacuse it is important to make exercise therapy a habit and to continue it in order to obtain the benefits of exercise, it is necessary to propose an exercise therapy program that is easy for patients to engage in.

We previously reported that the toe pinch force (TPF) of chronic hemodialysis patients with type 2 diabetes was significantly lower than that of chronic hemodialysis patients without type 2 diabetes [9]. The toes are closely involved in stabilizing the gait and balance function [10,11,12]. Decreased TPF increases the risk of falling while walking [13,14]. Because aerobic exercises such as walking are recommended for chronic hemodialysis patients with type 2 diabetes, exercise therapy may not be effective without improvement in TPF. Previously, we have performed four different types of toe resistance training designed for type 2 diabetic patients with reduced TPF and examined the effects of the training for two weeks [15]. The results showed that TPF improved in just two weeks. Furthermore, the ability of resistance training to affect TPF in just two weeks suggested that improved retention rates can be achieved through regular exercise therapy. Therefore, if the short-term effect of toe resistance training can be demonstrated in this study, it may indicate the possibility of a new exercise therapy option for chronic hemodialysis patients with type 2 diabetes in the future, and may even contribute to improving the continuation rate of exercise therapy. This study aimed to investigate the effectiveness of short-term toe resistance training in chronic hemodialysis patients with type 2 diabetes.

## 2. Materials and Methods

### 2.1. Study Design

This study was a prospective, parallel-group, single-blind, randomized controlled trial that included chronic hemodialysis patients with type 2 diabetes. Study participants were recruited from the in/outpatients of KKR Takamatsu Hospital (Takamatsu City, Kagawa Prefecture, Japan), Osafune Clinic (Setouchi City, Okayama Prefecture, Japan), and Obata Medical Clinic (Tsuyama City, Okayama Prefecture, Japan). The patients included in this study provided written informed consent. This study was approved by the Research Ethics Committee of Okayama Healthcare Professional University (number: 0030). The original research protocol is registered with the Japanese Clinical Trials Website (the University Hospital Medical Information Network: UMIN000026488).

### 2.2. Study Participants

**Figure 1:**
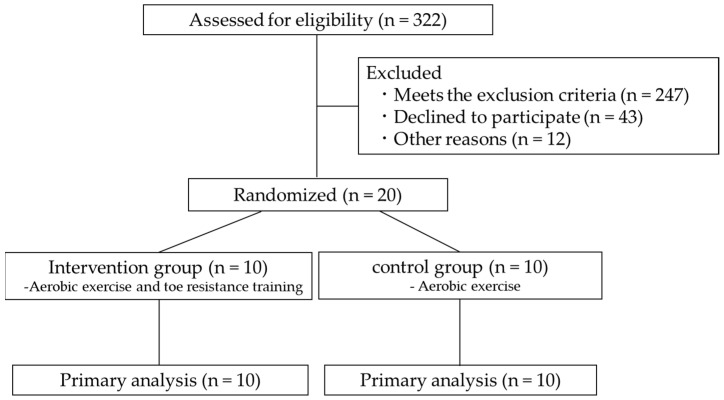
Flow diagram of the study, which included chronic hemodialysis patients with type 2 diabetes.

Twenty chronic hemodialysis patients with type 2 diabetes who underwent hemodialysis at three clinical research facilities from May to November 2021 were included in the study. Patients were randomly assigned to two groups using computer-generated random numbers: the intervention and control groups. (Figure 1). The exclusion criteria used were as follows: (1) patients with acute metabolic abnormalities, (2) acute or chronic orthopedic and/or cerebrovascular disease, (3) severe chronic diabetic complications (i.e., patients with severe sensory abnormalities or loss of sensation due to diabetic neuropathy, or patients with amputation, and patients who are blind due to diabetic retinopathy). (4) foot/toe deformity or foot edema, (5) significant limitation in daily activities, or (6) considered inappropriate for this study based on an interview and examination of medical records.

### 2.3. Sample Size

On the basis of our previous study [16], we set the effect size at 0.7 kg and the standard deviation at 0.47. Seventeen participants were required to have an 80% probability that the study would detect a TPF difference at a two-sided 5% level of significance. In addition, we assumed that 10% of the cases would be dropouts, resulting in a final total of 20 patients.

### 2.4. Exercise Intervention Program

**Figure 2:**
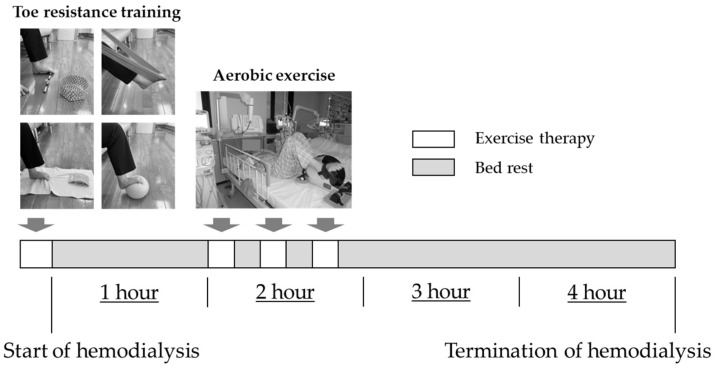
Exercise therapy protocol.

The protocol for exercise therapy is shown in Figure 2. The duration of the exercise therapy intervention was 2 weeks. The intervention group was instructed to perform aerobic exercise and toe resistance training, while the control group was instructed to perform aerobic exercise only. The aerobic exercise was performed using a bicycle ergometer (TERASU ERUGOIIIPLUS, Showa Denki Co. Ltd., Osaka, Japan) that can be used in the supine position. The exercise load was set at a moderate level. The exercise was performed for 30 min an hour after the start of dialysis therapy. Toe resistance training was performed prior to starting dialysis therapy. The four types of toe resistance training consisted of plantar flexion exercises of the ankle joint and flexion exercises of the toes using a towel, rubber ball, rubber tube, and small stick [16]. Toe resistance training consisted of three sets of 20 repetitions each for the left and right toes, with training intervals of at least 5 min. On days when dialysis therapy was not administered, the patients were instructed to perform exercise therapy at home. The intervention group was given toe resistance training and walking exercises, and the control group was given walking exercises. The walking exercise was set at a moderate intensity for 20 min per day.

### 2.5. Follow-Up after Exercise Intervention

The follow-up period was set at 6 months after the exercise intervention was completed. The intervention group was instructed to continue with four toe resistance training and walking exercise programs. The control group was instructed to continue with walking exercise program. During the follow-up period, both groups were periodically interviewed to determine whether they were continuing their exercise regimen, and any exercise-related consultation from the patients was addressed. At the end of the follow-up period, each parameter was evaluated.

### 2.6. Assessment of TPF

The TPF was evaluated using the same methods as in our previous study [17]. The Toe muscle dynamometer used was Checker-kun (Nisshin Sangyo Inc., Saitama, Japan). Measurement was taken with the participant in a chair. The posture for measurement was adjusted so that the angles of the hip and knee joints were each at 90°, and both upper limbs were crossed in front of the chest. The sensor of the TPF dynamometer was placed between the digitus primus and digitus secundus in a standby position. The patient was instructed to pinch the sensor with maximum force on the signal of the measurer. Measurements were taken twice each on the left and right side, with a 5 min rest between the first and second measurements. The average of the maximum values of the left and right toe muscle strength was then calculated.

### 2.7. Evaluation 

We collected data on sex; age; height; body weight; body mass index (BMI); heart rate (HR); systolic blood pressure (SBP); diastolic blood pressure (DBP); glycoalbumin (GA), fasting plasma glucose (FPG), albumin (Alb), hemoglobin (Hb), calcium (Ca), phosphorus (P), C-reactive protein (CRP), β2-microglobulin (β2MG), creatinine (CRE), and blood urea nitrogen (BUN) levels; estimated glomerular filtration rate (eGFR); normalized dialysis dose (Kt/Vurea); medications; duration of type 2 diabetes; and hemodialysis. Geriatric Nutritional Risk Index (GNRI) was used as an indicator of nutritional status [13]. Diet and drug therapy were investigated from medical records. The diagnoses of diabetic nephropathy was based on the guidelines of the Japan Diabetes Society [18]. Well-trained medical staff interviewed each patient to evaluate the International Physical Activity Questionnaire (IPAQ) short version [19], health-related quality of life (HRQOL), drinking, smoking, and exercise habits. HRQOL was evaluated using the EuroQol 5-dimensional (EQ-5D) questionnaire, which generates assessment scores across five dimensions of health, namely, mobility, self-care, usual activities, pain/discomfort, and anxiety/depression. Response in each dimension was divided into three categories; no problem, moderate problem, or extreme problem. Thereafter, a utility score was applied for this analysis according to our previous reports [20].

### 2.8. Outcome

The primary outcome of this study was to investigate the effectiveness of short-term toe resistance training. The secondary outcome was the rate of exercise retention 6 months after the end of the exercise intervention.

### 2.9. Data Analysis

All data were presented as mean ± standard deviation. First, the Shapiro–Wilk test was performed with the aim of confirming whether the clinical parameters evaluated were parametric or nonparametric data. Thereafter, the Mann–Whitney U test and χ^2^ test were used to compare the clinical data and TPF between the two groups at baseline. The Mann–Whitney U test, Wilcoxon signed-rank sum test, and χ^2^ test were used to compare the clinical data between the two groups before and after the exercise intervention and 6 months after the end of the exercise intervention, and *p* < 0.05 was considered statistically significant. All data were analyzed using JMP 12.2.0 software (SAS Institute, Cary, NC, USA).

## 3. Results

**Figure 3:**
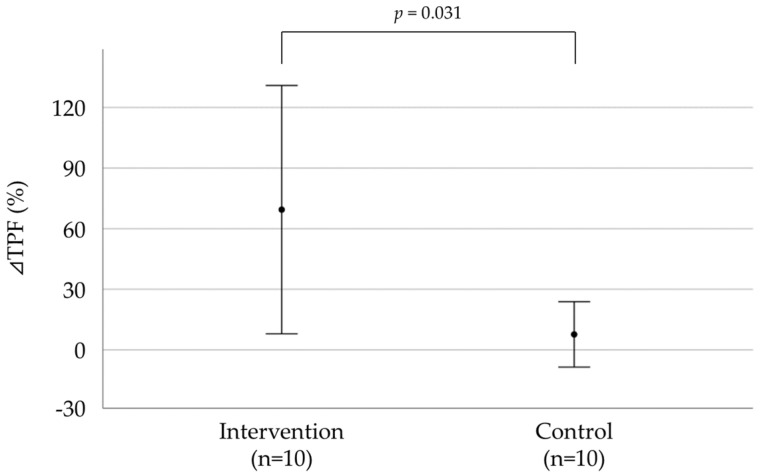
Comparison of the change in TPF after the exercise intervention program. ⊿ Represents the change in parameters. The Mann–Whitney U test was used for comparison between the two groups.

In total, 20 chronic hemodialysis patients with type 2 diabetes were recruited and randomly divided into intervention and control groups of 10 patients each. TPF in the intervention group was 2.09 ± 1.45 kg and that in control group was 2.46 ± 1.19 kg at baseline. Significant differences in DBP, CRE, and the duration of diabetes were noted between the groups. However, other parameters showed no significant differences between the groups (Table 1). The percent change in TPF and clinical data after the exercise intervention program are shown in Figure 3 and Table 2. The ⊿TPF (⊿ represents the change in the parameter) of the intervention group was significantly higher than that of the control group (69.5 ± 85.9 vs. 7.69 ± 22.6, *p* = 0.031). The ⊿DBP of the intervention group was significantly higher than that of the control group.

BMI, body mass index; SBP, systolic blood pressure; DBP, diastolic blood pressure; HR, heart rate; TPF, toe pinch force; GNRI, geriatric nutritional risk index; Alb, albumin; Hb, hemoglobin; CRP, C-reactive protein; P, phosphorus; Ca, calcium; β_2_MG, β_2_-microglobulin; BUN, blood urea nitrogen; CRE, creatinine; eGFR, estimated glomerular filtration rate; Kt/V, normalized dialysis dose; FPG, fasting plasma glucose; GA, glycoalbumin; IPAQ, International Physical Activity Questionnaire; EQ5D, EuroQol 5-dimensional questionnaire; GLP1, Glucagon-like peptide-1 receptor agonist; OHA, oral hypoglycemic agent; DPP-4, dipeptidyl peptidase-4 inhibitor; α-GI, α-glucosidase inhibitor

**Figure 4:**
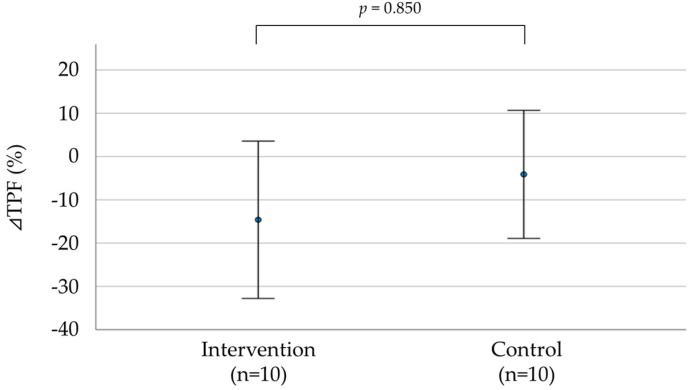
Comparison of the change in ⊿TPF 6 months after completion of the exercise intervention program. ⊿ Represents the change in parameters. The Mann–Whitney U test was used for comparison between the two groups.

Figure 4 and Table 3 show the percentage change in TPF and clinical parameters for 6 months after the end of the exercise intervention program. ⊿TPF, ⊿BMI, ⊿SBP, ⊿DBP, and ⊿HR were not significantly different between the groups. However, there was a significant difference in the rate of continuation of exercise therapy.

Table 4 shows the IPAQ and HRQOL at baseline and 6 months after exercise intervention. These were not significantly different between the groups.

## 4. Discussion

This study aimed to investigate the effectiveness of short-term toe resistance training in chronic hemodialysis patients with type 2 diabetes and investigate the continuation rate of toe resistance training. The results showed that TPF increased after only 2 weeks, demonstrating the effectiveness of toe resistance training. Furthermore, the results suggest that toe resistance training is an exercise that is easy to continue.

First, we discuss how TPF improved in a short period of time as a result of toe resistance training as we previously described [15]. As a muscle strengthening mechanism, muscle mass does not increase until about 3 weeks after the start of muscle strength training, and muscle strength increases first. This is known to involve neural factors [21]. Further, reportedly an increase in muscle mass is observed approximately 4 weeks after the start of muscle strength training [22]. Muscle mass was not assessed in this current study, and the mechanism of muscle strengthening by toe resistance training was not fully proven. However, the 2-week duration of the exercise therapy intervention suggests that, as in previous studies [15], neural factors may have influenced the improvement in TPF.

Next, we discuss the significantly higher rate of the continuation of exercise therapy in the intervention group, though there was no significant difference in TPF between the groups 6 months after the end of the exercise intervention. First, we describe TPF. In this study, exercise therapy was always conducted under the supervision of a physical therapist during the 2-week exercise intervention period; however, after the exercise intervention was completed, the study plan was changed to a non-supervised type because the patients were to continue exercising by themselves at home. If there were any questions or concerns about exercise therapy, the physical therapist would only respond to them on a case-by-case basis. Muscle strength increased if strength training was performed at 35% or more of the maximal muscle strength, and muscle strength decreased if strength training was performed at 20% or less [23]. The patients often asked questions and asked for advice regarding training methods. Therefore, it was suggested that the change in the exercise intervention method from supervised to unsupervised resulted in less accurate training and that failure to perform toe resistance training as instructed was the cause of the muscle weakness. We believe that the reason for the high rate of the continuation of exercise therapy in the intervention group was due to the fact that they were able to realize the benefits of toe resistance training in a short period of time. In addition, the four types of toe resistance training could be performed in a short time, and all the exercises could be performed easily in a sitting position, making the training safe and easy for the participants to engage in. The IPAQ showed no significant differences between the groups. In particular, more people in the intervention group were able to continue their exercise therapy, though they were less physically active than at baseline. In other words, the results suggest that toe resistance training may be a more effective training method than aerobic exercise for continuing exercise therapy. In light of the above, it is necessary, in the future, to examine whether the decline in TPF can be prevented by distributing leaflets and other materials describing toe resistance training methods to patients and periodically checking whether training is being performed appropriately.

We previously reported that TPF was lower in hemodialysis patients with type 2 diabetes than in hemodialysis patients without type 2 diabetes [9]. The loss of TPF is due to intrinsic muscle atrophy, which progressively inhibits the flexion and extension movements of the metatarsophalangeal joints. As a result, the toes become deformed, leading to hammer toe and claw toe. Since toe deformity is a risk factor for diabetic foot, intrinsic muscle atrophy must be prevented. If the disease progresses to diabetic foot, the risk of lower extremity amputation increases, and if it does occur, the prognosis for life is very poor. The life expectancy after lower extremity amputation in hemodialysis patients was reported to be 55% at 20 months and 81.3% without amputation; similarly, the 5-year and 10-year survival rates were 35% and 62.2%, respectively, and 15% and 33.3%, respectively [24]. Therefore, we believe that this study may be very informative as it provides a measure to prevent the development or progression of diabetic foot in hemodialysis patients with type 2 diabetes.

This study has a few limitations. First, although we were able to investigate the short-term effects of toe resistance training, we were not able to examine the long-term effects. Second, we did not include patients with type 1 diabetes or hemodialysis patients with non-diabetic conditions. In addition, the percentage of male hemodialysis patients is very high at 90%. Therefore, it is unclear whether the results of this study can be applied to hemodialysis patients with diseases other than type 2 diabetes or female hemodialysis patients.

## 5. Conclusions

In summary, four types of toe resistance training were found to improve TPF after only 2 weeks of training. Furthermore, the results suggest that toe resistance training may be a training program that easily leads to the continuation of exercise therapy. The results suggest that this type of muscle strength training may be a new exercise therapy for chronic hemodialysis patients with type 2 diabetes. Further studies are needed to determine the long-term effects of toe resistance training.

## Figures and Tables

**Table 1:** Clinical indicators of the intervention and control groups.

	Intervention	Control	*p*-Value
n	10	10	
Sex (male, n)	9	9	1.000
Age (years)	72.8 ± 9.2	69.5 ± 4.4	0.320
Height (cm)	163.8 ± 6.3	161.6 ± 4.9	0.385
Body weight (kg)	64.9 ± 9.6	59.0 ± 6.2	0.117
BMI (kg/m^2^)	24.1 ± 3	22.6 ± 2.2	0.208
SBP (mmHg)	135.3 ± 11.4	133.9 ± 15.7	0.822
DBP (mmHg)	65.5 ± 10.6	75.8 ± 10.1	0.039
HR (bpm)	65.2 ± 8.8	70.2 ± 7.4	0.187
Duration of hemodialysis (months)	48.9 ± 30.4	90.1 ± 71.9	0.112
TPF (kg)	2.09 ± 1.45	2.46 ± 0.38	0.540
GNRI score			
No risk, n (%)	1 (10)	3 (30)	0.512
Mild risk, n (%)	6 (60)	5 (50)	
Moderate risk, n (%)	3 (30)	2 (20)	
Alb (g/dL)	3.5 ± 0.2	3.7 ± 0.3	0.125
Hb (g/dL)	11.1 ± 0.8	10.6 ± 0.8	0.236
CRP (mg/dL)	0.12 ± 0.09	0.31 ± 0.37	0.136
P (mg/dL)	5.16 ± 1.60	5.51 ± 0.90	0.555
Ca (mg/dL)	8.65 ± 0.36	7.69 ± 1.97	0.147
β_2_MG (mg/dL)	23.1 ± 5.7	27.4 ± 8.1	0.180
BUN (mg/dL)	46.6 ± 21.7	58.2 ± 18.0	0.208
CRE (mg/dL)	7.2 ± 3.1	10.0 ± 2.0	0.026
eGFR (mL/min/1.73 m^2^)	5.96 ± 3.07	4.76 ± 1.26	0.267
Kt/V	1.46 ± 0.22	1.47 ± 0.18	0.885
FPG (mg/dL)	125.1 ± 35.8	129.0 ± 25.2	0.781
GA (%)	18.9 ± 2.9	21.9 ± 4.5	0.090
Duration of diabetes (months)	293.5 ± 132.3	169.0 ± 113.1	0.036
IPAQ (kcal/day)	73.9 ± 58.4	79.4 ± 133.5	0.907
EQ5D score	0.85 ± 0.16	0.84 ± 0.15	0.812
Exercise habit, n (%)	4 (40)	1 (10)	0.303
Drinking habit, n (%)	2 (20)	1 (10)	1.000
Smoking habit			
Current smoker, n (%)	1 (10)	3 (30)	0.501
Previous smoker, n (%)	7 (70)	5 (50)	
Non-smoker, n (%)	2 (20)	2 (20)	
Diet therapy, n (%)	5 (50)	5 (50)	1.000
Drug therapy			
GLP-1, n (%)	1 (10)	1 (10)	0.159
GLP-1 + OHA (Glinide), n (%)	0	1 (10)	
OHA			
DPP-4, n (%)	6	1	
α-GI, n (%)	1	0	
Glinide, n (%)	0	1	
Insulin, n (%)	1 (10)	2 (20)	
None, n (%)	1 (10)	4 (40)	

**Table 2:** Comparison of the changes in clinical characteristics after the exercise intervention program.

	Intervention (n = 10)	Control (n = 10)	*p*-Value
⊿BMI	0.29 ± 0.84	0.57 ± 1.34	0.579
⊿SBP	−0.48 ± 6.23	−9.07 ± 14.83	0.109
⊿DBP	11.84 ± 14.51	−10.01 ± 13.63	0.004
⊿HR	−1.64 ± 8.82	1.49 ± 13.94	0.556

BMI, body mass index; SBP, systolic blood pressure; DBP, diastolic blood pressure; HR, heart rate. ⊿ Represents the change in parameters.

**Table 3:** Comparison of the changes in clinical characteristics and continuation of exercise therapy 6 months after completion of exercise intervention program.

	Intervention (n = 10)	Control (n = 10)	*p*-Value
⊿BMI	−0.18 ± 4.84	−0.08 ± 3.72	0.970
⊿SBP	−0.63 ± 11.74	18.3 ± 30.33	0.070
⊿DBP	−6.44 ± 17.47	11.52 ± 24.55	0.121
⊿HR	2.28 ± 14.55	5.44 ± 18.28	0.910
Continuation of exercise therapy, n (%)	6 (60)	1 (10)	0.019

Values are presented as the mean ± SD. BMI, body mass index; SBP, systolic blood pressure; DBP, diastolic blood pressure; HR, heart rate. ⊿ Represents the change in parameters.

**Table 4:** Comparison of IPAQ and HRQOL at baseline and 6 months after exercise intervention.

	Intervention (n = 10)			Control (n = 10)		
	Baseline	Follow-Up	*p*-Value	Baseline	Follow-Up	*p*-Value
IPAQ (kcal/day)	314.9 ± 737.5	249.0 ± 335.9	1.000	79.4 ± 133.5	117.3 ± 241.0	0.844
EQ5D score	0.853 ± 0.162	0.857 ± 0.126	0.906	0.836 ± 0.149	0.832 ± 0.113	0.938

Value are presented as mean ± SD. IPAQ, International Physical Activity Questionnaire; EQ5D, EuroQol 5-dimensional questionnaire.

## Data Availability

Not applicable.

## References

[1] Avesani C.M., Trolonge S., Deléaval P., Baria F., Mafra D., Faxén-Irving G., Chauveau P., Teta D., Kamimura M.A., Cuppari L. (2012). Physical activity and energy expenditure in hemodialysis patients: An international survey. Nephrol. Dial. Transplant..

[2] Painter P. (2005). Physical functioning in end-stage renal disease patients: Update 2005. Hemodial. Int..

[3] Sterky E., Stegmayr B.G. (2005). Elderly patients on hemodialysis have 50% less functional capacity than gender-and age- matched healthy subjects. Scand. J. Urol. Nephrol..

[4] Pu J., Jiang Z., Wu W., Li L., Zhang L., Li Y., Liu Q., Ou S. (2019). Efficacy and safety of intradialytic exercise in hemodialysis patients: A systematic review and meta-analysis. BMJ Open.

[5] Matsuzawa R., Hoshi K., Yoneki K., Harada M., Watanabe T., Shimoda T., Yamamoto S., Matsunaga A. (2017). Exercise training in elderly people undergoing hemodialysis: A systematic review and meta-analysis. Kidney Int. Rep..

[6] Sheng K., Zhang P., Chen L., Cheng J., Wu C., Chen J. (2014). Intradialytic exercise in hemodialysis patients: A systematic review and meta-analysis. Am. J. Nephrol..

[7] Segura-Ortí E. (2010). Exercise in hemodialysis patients: A systematic review. Nefrologia.

[8] The Japanese Society for Dialysis Therapy. https://docs.jsdt.or.jp/overview/index2019.html.

[9] Kataoka H., Miyatake N., Matsuda N., Hikasa Y., Kitayama N., Nagai S., Tanaka S. (2021). The association between chronic hemodialysis and toe pinch force in Japanese patients: A cross-sectional study. Healthcare.

[10] Endo M., Ashton-Miller J.A., Alexander N.B. (2002). Effects of age and gender on toe flexor muscle strength. J. Gerontol. A Biol. Sci. Med. Sci..

[11] Menz H.B., Morris M.E., Lord S.R. (2005). Foot and ankle characteristics associated with impaired balance and functional ability in older people. J. Gerontol. A Biol. Sci. Med. Sci..

[12] Mann R., Inman V.T. (1964). Phasic activity of intrinsic muscles of the foot. J. Bone Jt. Surg. Am..

[13] Menz H.B., Morris M.E., Lord S.R. (2006). Foot and ankle risk factors for falls in older people: A prospective study. J. Gerontol. A Biol. Sci. Med. Sci..

[14] Mickle K.J., Munro B.J., Lord S.R., Menz H.B., Steele J.R. (2009). ISB Clinical Biomechanics Award 2009: Toe weakness and deformity increase the risk of falls in older people. Clin. Biomech..

[15] Kataoka H., Miyatake N., Murao S., Tanaka S. (2018). A randomized controlled trial of short-term toe resistance training to improve toe pinch force in patients with type 2 diabetes. Acta Med. Okayama.

[16] Kataoka H., Miyatake N., Kitayama N., Murao S., Tanaka S. (2017). A pilot study of short-term toe resistance training in patients with type 2 diabetes mellitus. Diabetol. Int..

[17] Kataoka H., Miyatake N., Kitayama N., Murao S., Tanaka S. (2017). Toe pinch force in male type 2 diabetes mellitus patients. Acta Med. Okayama.

[18] Tajima N., Noda M., Origasa H., Noto H., Yabe D., Fujita Y., Goto A., Fujimoto K., Sakamoto M., Haneda M. (2015). Evidence-based practice guideline for the treatment for diabetes in Japan 2013. Diabetol. Int..

[19] Craig C.L., Marshall A.L., Sjöström M., Bauman A.E., Booth M.L., Ainsworth B.E., Pratt M., Ekelund U., Yngve A., Sallis J.F. (2003). International physical activity questionnaire: 12-country reliability and validity. Med. Sci. Sports Exerc..

[20] Kataoka H., Miyatake N., Ichikawa H., Arakawa Y., Mori Y. (2019). Sub-analysis of the prevalence of locomotive syndrome and its relationship with health-related quality of life in patients with obstructive sleep apnea syndrome as classified by age and sex. Sleep Biol. Rhythms.

[21] Kraemer W.J., Fleck S.J., Evans W.J. (1996). Strength and power training physiological mechanism of adaptation. Exerc. Sport Sci. Rev..

[22] Moritani T., deVries H.A. (1980). Potential for gross muscle hypertrophy in older men. J. Gerontol..

[23] Muller E.A. (1952). Training muscle strength. Ergonomics..

[24] Sánchez Perales M.C., García Cortés M.J., Borrego Utiel F.J., Viedma G., Gil J.M., Pérez del Barrio P., Borrego Hinojosa J., Liébana A., Pérez Bañasco V. (2005). Incidence and risk factors for non-traumatic lower extremity amputation in hemodialysis patients. Nefrologia.

